# Association of caregiver demographic variables with neuropsychiatric symptoms in Alzheimer’s disease patients for distress on the Neuropsychiatric Inventory (NPI)

**DOI:** 10.1590/S1980-57642009DN20300009

**Published:** 2008

**Authors:** Cláudia Godinho, Analuiza Camozzato, Renata Kochhann, Márcia Lorena Fagundes Chaves

**Affiliations:** 1 Dementia Clinic, Neurology Service, Hospital de Clínicas de Porto Alegre.; 2 Medical Sciences Post-Graduation Course, UFRGS School of Medicine.; 3 Internal Medicine Department, UFRGS School of Medicine

**Keywords:** neuropsychiatric symptoms, Alzheimer’s disease, distress, caregiver, Brazil

## Abstract

Behavioral symptoms are frequently observed in Alzheimer’s disease patients and are associated to higher distress for patients and caregivers, early institutionalization, worst prognosis and increased care.

**Objectives:**

The objective of the present study was to evaluate the frequency of neuropsychiatric symptoms in a sample of Alzheimer’s disease patients and to analyze association between caregiver demographic characteristics and patient symptoms.

**Methods:**

Sixty Alzheimer’s disease patients (NINCDS-ADRDA) and their caregivers were consecutively included in the investigation by the Dementia Outpatient clinic of Hospital de Clínicas de Porto Alegre. The Neuropsychiatric Inventory (NPI) was applied to evaluate behavioral symptoms and their impact upon caregivers. Age, sex, educational attainment, relationship to the patient, and time as caregiver were obtained from all caregivers.

**Results:**

Apathy was the symptom responsible for the highest distress level, followed by agitation and aggression. A significant correlation between total severity NPI and distress NPI was observed. None of the caregiver demographic data showed association to distress. The most frequent symptoms were apathy and aberrant motor behavior. Patients’ relatives also considered apathy as the most severe symptom, followed by depression and agitation.

**Conclusions:**

Apathy was the most frequent and severe neuropsychiatric symptom. No relationship between caregiver demographic characteristics and distress was observed.

## Abstract (pt)

Sintomas comportamentais são freqüentemente observados nos pacientes com doença de Alzheimer e estão associados a maior desgaste para pacientes e cuidadores, institucionalização precoce, pior prognóstico e mais cuidado.

**Objetivos:**

O objetivo do presente estudo foi avaliar freqüência de sintomas neuropsiquiátricos em uma amostra de pacientes com doença de Alzheimer e analisar a associação entre características demográficas do cuidador e sintomas do paciente.

**Métodos:**

Um total de 60 pacientes com doença de Alzheimer (NINCDS-ADRDA) e seus cuidadores foram consecutivamente incluídos na investigação provenientes do Ambulatório de Demência do Hospital de Clínicas de Porto Alegre. O Inventário Neuropsiquiátrico (INP) foi aplicado para avaliar sintomas comportamentais e seu impacto sobre os cuidadores. Idade, sexo, educação, parentesco com o paciente e tempo como cuidador foram obtidos dos cuidadores.

**Resultados:**

Apatia foi o sintoma responsável pelo maior nível de desgaste, seguido por agitação e agressão. Uma correlação significativa entre escore total do INP e desgaste foi observada. Nenhuma das variáveis demográficas do cuidador mostrou associação com desgaste. Os sintomas mais freqüentes foram apatia e comportamento motor aberrante. Os familiares do pacientes também consideraram apatia como sintoma mais grave seguido por depressão e agitação.

**Conclusões:**

Apatia foi o sintoma neuropsiquiátrico mais freqüente e grave. Nenhuma relação entre as variáveis demográficas do cuidador e desgaste foi observada.

Behavioral symptoms constitute part of the clinical presentation of Alzheimer’s disease (AD) and may occur in any phase of the disease. Some of the most frequent symptoms include agitation, apathy, depression, disinhibition, sleep disturbances, delusions, eating abnormalities, hallucinations, aggression, and personality changes.^1^

The prevalence of the neuropsychiatric symptoms in AD ranges from 25% to 80% where this variation depends on the study methodology.^2-7^ The most common symptom has proved to be apathy, affecting 72% of patients interviewed using the Neuropsychiatric Inventory (NPI).^3-7^

Neuropsychiatric symptoms in AD are associated with significant distress for patients and caregivers, higher costs and poor prognosis.^2,8-10^ These symptoms are also responsible for the early institutionalization of patients.^11^

Pharmacological and behavioral treatment of non-cognitive symptoms of AD has been the target of extensive scientific investigation.^1,12^ Reliable and valid tools to measure such symptoms are necessary to allow proper assessment in clinical trials and for the appropriate approach to be adopted in clinical practice.

The Neuropsychiatric Inventory was developed and validated by Cummings (1994)^13^ with the objective of gathering information on the presence, frequency, and severity of behavioral symptoms in dementia patients. The other goal of this tool is to evaluate the impact of each symptom domain on caregivers.^14^ NPI encompasses twelve domains (apathy, delusions, hallucinations, agitation/aggression, depression/dysphoria, anxiety, elation/euphoria, aberrant motor behavior, night-time behavior, irritability/lability, disinhibition, and appetite/eating abnormalities). Validated versions of NPI are available in several languages including Greek,^15^ the Yoruba dialect in Nigeria,^16^ Danish,^17^ Japanese,^18^ Korean,^19^ Spanish,^20^ Chinese,^21^ and Brazilian Portuguese.^22^ A Brazilian study has shown good reliability and internal consistency of NPI, along with a similar profile of behaviors to those observed in other countries.^22^

The distress NPI (NPI-D) was developed to measure emotional and psychological burden on caregivers under each NPI symptom domain. Besides information on the presence, frequency, and severity of behavioral symptoms, caregivers also score the distress induced by symptoms. A 6-point scale is used for the scoring, encompassing the following scores: 0, not at all distressing; 1, minimally distressing; 2, mildly distressing; 3, moderately distressing; 4, severely distressing; and 5, very severely or extremely distressing.^14^ Validity of the NPI-D was assessed by comparing it to the Relatives’ Stress Scale (RSS), where significant correlation, adequate test-retest and interrater reliability were observed.^4^

The main hypothesis of this investigation was that some caregiver demographic characteristics would influence perception of distress whereby women, less educated persons, children of patients were expected to experience higher levels of distress. The objective of the present study was to evaluate frequency of neuropsychiatric symptoms in a sample of Alzheimer’s disease patients and to analyze association between caregiver demographic characteristics and patient symptoms.

## Methods

Sixty consecutive patients and their caregivers who were seen for a 10-month period in the Dementia outpatient clinic of the Hospital de Clínicas de Porto Alegre and that fulfilled the *National Institute of Neurological and Communicative Disorders and Stroke-Alzheimer’s Disease and Related Disorders Association* (NINCDS-ADRDA) criteria for probable Alzheimer’s disease were included in the study.^23^ Patients underwent a full routine evaluation, including: the Mini Mental State Examination,^24,25^ a battery of cognitive tests, the CDR scale,^26,27^ a neurological examination, screening laboratory tests, and a brain CT. After the diagnosis definition, the NPI was applied for the assessment of behavioral symptoms and their impact on caregivers.

Study variables for caregivers were age, sex, educational attainment, level of relationship to the patient, and length of time as caregiver (in months). Demographic and clinical data for patients and caregivers are displayed in Table 1.

**Table 1:** Clinical and demographic data of patients and caregivers.

Variables	AD patients	Caregivers
Gender N (%)		
Female	45 (75%)	50 (83%)
Male	15 (25%)	10 (17%)
CDR N (%)		
1	12 (24%)	-
2	14 (29%)	-
3	23 (47%)	-
Relationship to the patient		
Spouse	-	11 (18.3)
Patient's child	-	41 (68.3)
Other relative	-	4 (6.7)
Non relative	-	4 (6.7)
Age *	77.9±8.4	52.4±12.8
Years of education *	4.8±3.8	9.1±3.7
Time as caregiver (months)*	-	48.8±36

* mean±SD

According to the sample size calculation, 52 patients and their caregivers would be sufficient for the present study, given a minimum symptom frequency of 46%28 using an alpha error of 5% and beta error of 20% determined by the OpenEpi version2 Program.

The study was approved by the Ethics Committee for Medical Research at Hospital de Clínicas de Porto Alegre. Patients and their proxies signed an informed consent before being enrolled onto the study.

### Data analysis

Descriptive statistics (mean, SD and frequency) were calculated for demographic data, symptoms of NPI, and CDR. Spearman’s rho correlation coefficients were estimated for age, education, time as caregiver, distress NPI and total severity NPI. The comparison of distress scores between the CDR global scores (≤2 and 3), sex and relationship to the patient was tested with the non-parametric Mann-Whitney U test. The statistical analysis was performed using the Statistical Package for the Social Sciences (SPSS 14).

## Results

**Figure 1:**
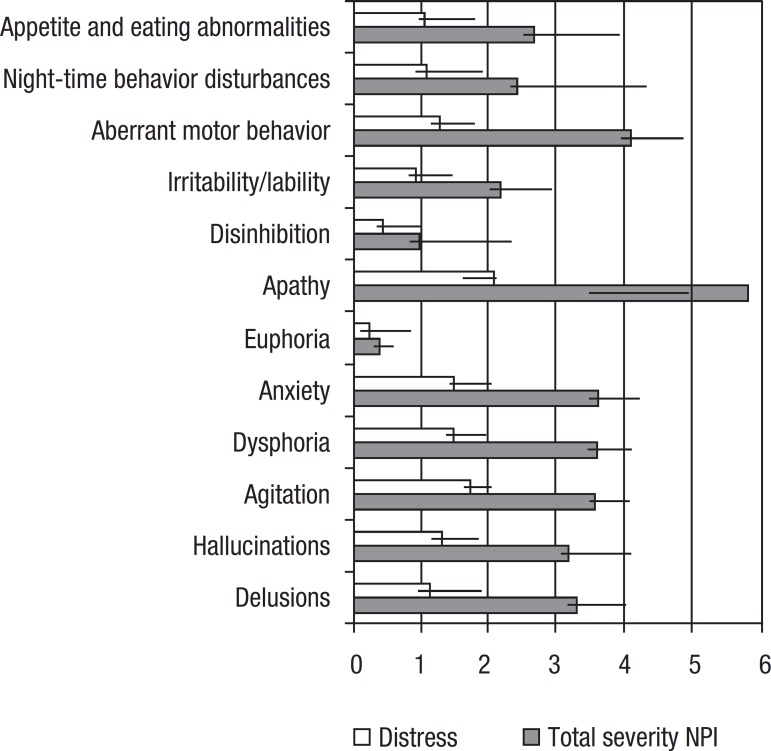
Mean and SD for distress and total severity NPI (frequency × severity) of each neuropsychiatric symptom.

Caregiver distress caused by each neuropsychiatric symptom is presented in Table 2. Apathy caused the highest level of distress followed by agitation and aggression (Figure 1).

**Table 2:** Association between caregiver distress level and patient symptom domains.

Symptom domains	Not present	Minimally/mildly	Moderately	Severely/very severely
Delusions	23 (54%)	4 (7%)	10 (17%)	13 (22%)
Hallucinations	36 (60%)	6 (10%)	7 (12%)	11 (18%)
Agitation/Aggression	29 (48%)	7 (12%)	9 (15%)	15 (25%)
Depression	30 (50%)	10 (17%)	10 (17%)	10 (17%)
Anxiety	33 (55%)	8 (13%)	6 (10%)	13 (22%)
Euphoria	56 (93%)	1 (2%)	1 (2%)	2 (3%)
Apathy	23 (38%)	10 (17%)	8 (13%)	19 (32%)
Disinhibition	50 (83%)	6 (10%)	3 (5%)	1 (2%)
Irritability/Lability	41 (70%)	5 (8%)	6 (10%)	7 (12%)
Aberrant motor behavior	37 (62%)	5 (8%)	7 (12%)	11 (18%)
Night-time behavior disturbances	43 (72%)	2 (3%)	2 (3%)	13 (22%)
Appetite and eating abnormalities	42 (70%)	3 (5%)	7 (12%)	8 (13%)

**Figure 2:**
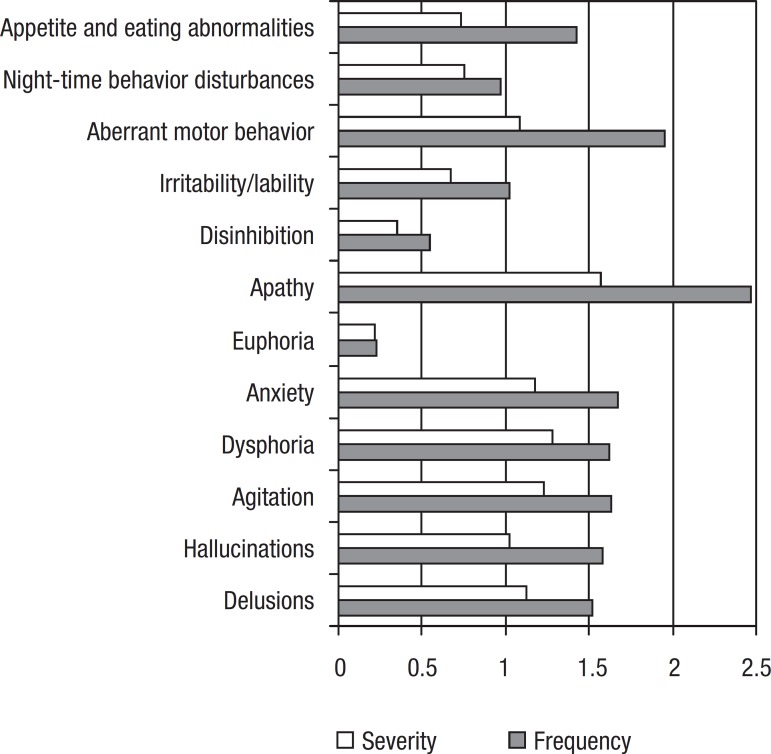
Frequency and severity of symptom domain

Most frequent symptoms were apathy and aberrant motor behavior. Apathy was also considered by caregivers as the most severe symptom followed by depression and agitation (Figure 2).

No significant correlation between distress and caregiver demographic variables was observed. Only total severity NPI score presented moderate correlation with distress (Table 3). No difference in distress scores was observed between CDR categories (p=0.079), caregiver sex (p=0.976) and relationship of the caregiver to the patient (p=0.622) (Mann Whitney U test). No difference in total severity NPI scores was found between CDR categories (p=0.681) (Mann Whitney U test).

**Table 3:** Correlation of distress NPI with caregiver demographic characteristics and total severity NPI.

Variables	Spearman's Rho Correlation coefficient	P value
Age	-0.125	0.351
Education	-0.229	0.084
Time as caregiver	-0.170	0.205
Total severity NPI	0.731	0.000

Correlation between frequency, severity and total severity, and caregiver distress for each symptom domain was significant (p<0.001; moderate to strong) (Table 4).

**Table 4:** NPI Component scores and relationship to NPI-Distress score.

Symptoms	Distress Mean±SD	Frequency Mean±SD	Rho*	Severity Mean±SD	rho*	Total severity Mean±SD	rho*
Delusions	1.56±1.9	1.52±1.6	0.791	1.13±1.2	0.819	3.28±3.9	0.819
Hallucinations	1.3±1.8	1.58±1.7	0.774	1.02±1.09	0.823	3.18±4.1	0.823
Agitation/Aggression	1.73±1.84	1.63±1.5	0.667	1.23±1.1	0.706	3.57±4.0	0.676
Dysphoria/Depression	1.47±1.71	1.62±1.5	0.719	1.28±1.18	0.792	3.58± 4.1	0.780
Anxiety	1.47± 1.84	1.67±1.65	0.726	1.18±1.18	0.804	3.5±2.3	0.784
Euphoria/Elation	0.22±0.9	0.23±0.61	0.647	0.22±0.613	0.667	0.37±1.28	0.669
Apathy/Indifference	2.08±1.95	2.5±1.8	0.654	1.6±1.25	0.776	5.83±5.0	0.753
Disinhibition	0.42±1.03	0.55±1.18	0.867	0.35±0.73	0.859	0.97±2.37	0.867
Irritability/Lability	2.5±1.4	2.6±1.0	0.950	1.5±0.7	0.952	4.1±2.8	0.953
Aberrant motor behavior	1.28±1.73	1.95±1.57	0.702	0.75±1.18	0.741	4.08±4.82	0.747
Night-time behavior	1.08±1.82	0.97±1.57	0.913	0.75± 1.18	0.954	2.42±4.28	0.939
Appetite/eating abnormalities	1.05±1.72	1.43±1.82	0.760	0.73±1.02	0.798	2.67±3.89	0.788

* Spearman rank correlation coefficients (rho) between NPI frequency, severity, total severity scores and NPI-Distress scale score.

## Discussion

The present study was developed to evaluate the frequency of neuropsychiatric symptoms in a sample of Alzheimer’s disease patients and to analyze association between caregiver demographic variables and distress caused by patient symptoms. The most frequent symptom was apathy as shown in several previous studies.^2,15,18,29-32^

Apathy was also the symptom which caused most distress in caregivers. No association was found between distress and caregiver sex, age and educational attainment, degree of relationship to the patient or time as caregiver. These results showed that distress was independent from the evaluated demographic characteristics. Caregiver distress was strongly associated to neuropsychiatric symptoms because distress was rated in relation to symptoms. The correlation of total severity NPI with the total distress NPI scores has previously been demonstrated to be strong but less homogeneous across the symptoms domains.^14^ A recent study on the correlation between caregiver stress and patient clinical characteristics also showed that stress was associated to psychiatric symptoms.^33^ On the other hand, functional impairment degree, severity of cognitive deficit, dementia symptoms, history duration, length of time as caregiver, caregiver living with the patient, and having a previous diagnosis, also correlated with distress. These conflicting findings have been partially linked to cultural differences. However, it is possible that other factors such as study design, concomitant pharmacological treatment or non-pharmacological intervention could also play a part.

Distress NPI did not differ for severity of dementia because total severity NPI was similar across CDR categories. Distress NPI is measured, as outlined above, for each neuropsychiatric symptom. The CDR scale measures cognitive and functional impairment but not neuropsychiatric symptoms. In this sense, severity of dementia by CDR is unable to evaluate distress caused by behavioral symptoms.

As observed earlier, caregivers who are patient relatives presented worst physical and psychological health than patient relatives who do not perform this role.^34-38^ Since our sample was composed of 94% of caregiver relatives, this concern is highly relevant. To prevent these health problems the identification of behavioral symptoms and other determinant factors of distress impact, often referred to as ‘burden of care’, is required.

The study limitations were the small variability of caregiver demographic variables, the inclusion of patients attending an outpatient clinic, which could have prevented the inclusion of more severe cases of dementia, and the fact that the clinic is a reference center for dementia care could have led to a different profile of behavioral symptom frequency and severity compared to those observed in non-specialized clinics. However, the strength of this study was its stringent selection of AD patients based on contemporary criteria, and the use of good quality caregivers to yield reliable information.

Interventions to reduce caregiver distress should take into account this data in a bid to identify those caregiver and patient characteristics related to higher stress. The finding of apathy as the most frequent symptom causing most distress is an issue for future investigation since this has been demonstrated repeatedly in the literature but has not yet been sufficiently explained.
